# Multi-organ Immune-Related Adverse Event Is a Risk Factor of Immune Checkpoint Inhibitor-Associated Myocarditis in Cancer Patients: A Multi-center Study

**DOI:** 10.3389/fimmu.2022.879900

**Published:** 2022-07-18

**Authors:** Xiaohong Xie, Liqiang Wang, Yingqing Li, Yan Xu, Jianhui Wu, Xinqing Lin, Wen Lin, Qicong Mai, Zhanhong Chen, Jiexia Zhang, Zhanhong Xie, Yinyin Qin, Ming Liu, Mingjun Lu, Bihui Luo, Chengzhi Zhou

**Affiliations:** ^1^ Laboratory of Respiratory Disease, National Clinical Research Center for Respiratory Disease, Guangzhou Institute of Respiratory Health, the First Affiliated Hospital of Guangzhou Medical University, Guangzhou, China; ^2^ College of Life Science, Henan University, Kaifeng, China; ^3^ Department of Emergency, Outpatient of Cardiovascular, State Key Laboratory of Oncology in South China, Collaborative Innovation Center of Cancer Medicine, Sun Yat-sen University Cancer Center, Guangzhou, China; ^4^ Department of Respiratory and Critical Care Medicine, Peking Union Medical College Hospital, Chinese Academy of Medical Sciences and Peking Union Medical College, Beijing, China; ^5^ Department of Medical Oncology, Cancer Hospital of Shantou University Medical College, Shantou, China; ^6^ Department of Interventional Radiology, Cancer Center, Guangdong Provincial People’s Hospital, Guangdong Provincial Academy of Medical Sciences, Guangzhou, China; ^7^ Department of Oncology, The Third Affiliated Hospital of Sun Yat-sen University, Guangzhou, China; ^8^ Department of Cardiology, The First Affiliated Hospital of Guangzhou Medical University, Guangzhou, China

**Keywords:** myocarditis, immune checkpoint inhibitors, immune-related adverse events, prognosis, multi-organ irAEs

## Abstract

**Background and Objective:**

Immune checkpoint inhibitor (ICI)-associated myocarditis is a fatal immune-related adverse events (irAEs), which is prone to affecting multiple organ systems. Multi-organ irAEs have not been fully studied in ICI-associated myocarditis. Therefore, we aimed to explore the impact of multi-organ irAEs on ICI myocarditis in terms of clinical features, treatment, and prognosis.

**Methods:**

This was a retrospective study. The clinical data of ICI myocarditis patients were collected from 6 hospitals in China. The risk factors and characteristics of pure myocarditis and multi-organ irAEs were analyzed. The overall survival (OS) after myocarditis was analyzed and univariate and multivariate regression analysis were performed.

**Results:**

A total of 46 patients were analyzed in this study. Multi-organ irAEs were common (30/46, 65.2%) and prone to severe heart failure. The severe myocarditis was observed in 32 patients (69.6%). When myocarditis occurred, neutrophil to lymphocyte ratio, C-reactive protein, lactate dehydrogenase, interleukin (IL)-6, IL-10, creatine kinase, MB isoenzyme of creatine kinase, and brain natriuretic peptide increased from baseline, but absolute lymphocyte count decreased. Thymoma (B2/B3) was a risk factor for multi-organ irAEs. Heart failure and myocarditis were more severe in patients with multi-organ irAEs and require early corticosteroid therapy (<24 hours). Univariate analysis showed that age ≥ 60 years, myocarditis (grade 3-4), heart failure (grade 3-4), multi-organ irAEs, and severe myocarditis were associated with OS after myocarditis. After adjusting for other factors, heart failure (grade 3-4) was an independent risk factor for immune-related myocarditis (HR: 6.655, 95% CI: 1.539-28.770, p=0.011).

**Conclusion:**

Patients with ICI-associated myocarditis had multi-organ irAEs with a high incidence of severe myocarditis, mortality, and poor prognosis. Thymoma was prone to those patients with multiple organs involvement. Patients could benefit from early corticosteroid intervention. Heart failure (grade 3-4) was an independent risk factor for OS after myocarditis.

## Introduction

In recent years, many patients have benefited from immune checkpoint inhibitors (ICIs). However, immunotherapy is a double-edged sword because immune-related adverse events (irAEs) can lead to fatal outcomes (1, 2). IrAEs can occur in any organ, and multi-organ involvement is rare. Ganessan et al. reported that among 1548 patients, 424 patients (27%) had irAEs, of which only 84 patients (5.4%) had multi-organ irAEs (3). In another study comprising 623 patients, 148 patients (24%) developed a single irAE, whereas 58 (9.3%) exprienced multi-system irAEs (4).

ICI-associated myocarditis is a kind of irAEs with a very low incidence. A previous study pooled 31321 patients receiving ICIs, of which 122 (0.38%) developed ICI myocarditis, with 18 (0.09%) developed severe myocarditis (5). Mahmood et al. reported the incidence of ICI myocarditis was 1.14%, and myocarditis was more likely to occur during combined chemotherapy (34% versus 2%; P<0.001) (6). The combination use of ICI may even induce fulminant myocarditis (7). However, ICI myocarditis is a fatal AE with a lethality rate of 39.7%-50% (5, 8). Because of the low incidence, limited studies have explored the risk factors, pathogenesis, treatment and prognosis of ICI myocarditis. A retrospective study involving eight centers showed that diabetes, obstructive sleep apnea, high body mass index (BMI), and receiving angiotensin receptor blockers are risk factors for ICI myocarditis. Another study showed that a history of heart failure and acute coronary syndrome are also high-risk factors for ICI myocarditis (6, 9). In addition, a study found that patients with myocarditis have a decrease in ALC and an increase in neutrophil to lymphocyte ratio (NLR), and a decrease in absolute lymphocyte count (ALC) >35% or an increase in NLR >100% can predict subsequent major adverse cardiac events (MACE). All these suggest that the decrease of ALC and the increase of NLR contribute to the diagnosis and risk stratification of patients with myocarditis (10). In addition, Zlotoff et al. founded that the extended duration of QRS leads to an increased risk of MACE (11).

Single-system irAEs have been widely reported, but there are few reports on the clinical patterns and survival effects of irAEs involving multiple organ systems. Previous studies on non-small cell lung cancer (NSCLC) and other tumors have found that the occurrence of some irAEs such as thyroiditis and checkpoint inhibitor pneumonitis (CIP) is associated with improved survival outcomes (12, 13). Ganessan et al. founded that multi-organ irAEs were associated with improved overall survival compared with no irAEs or single-organ irAE (3). Previous studies have shown that 47% of 55 ICI myocarditis patients have multi-organ irAEs, indicating that heart-related irAEs are more likely to be affected by multiple organ systems (10). However, multi-organ irAEs have not been fully studied in patients with myocarditis. In China, there are less researches on the risk factors, treatment, and prognostic factors of ICI myocarditis. In order to evaluate the role of multiple organs involvement in ICI myocarditis, we collected data from 6 centers in China to explore the impact of multi-organ irAEs on ICI myocarditis in terms of clinical features, treatment, and prognosis.

## Patients and Methods

### Patients and Diagnostic Criteria

A systematic search was carried out in 6 medical centers in China to identify tumor patients who developed myocarditis after receiving immune checkpoint inhibitors (programmed death [PD]-1/ligand 1 [L1] or cytotoxic T lymphocyte-associated antigen-4 [CTL4]) between January 2018 and August 2021 (**Supplementary Table 1**). Informed consent and review of the study were waived by the Institutional Review Board of each hospital. Adverse events occurred while patients were on ICIs or within 30 days after the last dose were attributed to ICIs and were adjudicated as irAEs. These events were organ specific, including cardiovascular, lung, skin, liver and gallbladder and pancreas, endocrine, gastrointestinal, rheumatism, nervous system, laboratory abnormalities and other miscellaneous organ specificities. Cardiologists further reviewed the course of the included patients to determine that the occurrence of myocarditis was attributable to ICIs. Patients with missing clinical data, not receiving ICIs, and other causes of myocarditis were excluded. Diagnosis of ICI myocarditis was performed in either of two ways: 1) histopathology, or 2) clinically suspected myocarditis (according to the European Society of Cardiology guidelines) (14). The diagnosis of other irAEs was based on pathological evidence or multidisciplinary adjudication, or the irAE-based treatment improved clinical outcomes excluding alternative diagnosis (15). Multi-organ irAEs were defined as irAEs that affected more than one organ or system, other than ICI myocarditis.

### Data Collection

Data were retrospectively obtained from medical records, including demographic characteristics (age, gender, risk factors related to heart disease, heart-related risk factors), characteristics and treatment information of tumor (cancer type, clinical stage, tumor treatment information), characteristics and prognosis of myocarditis (symptoms, grade of heart failure, grade of myocarditis, survival information, cause of death). The grade of heart failure was based on New York Heart Association (NYHA) classification. The grade of ICI myocarditis was based on the American Society of Clinical Oncology Clinical Practice Guideline (15). Severe ICI myocarditis is defined as grade 3-4 myocarditis. Patients were categorized into low-dose (<500mg/day) and high-dose (>500mg/day) groups based on initial methylprednisolone-equivalent administered on the first day of treatment. The timing of corticosteroid therapy is defined as the time from the onset of heart-related symptoms or asymptomatic but abnormal cardiac examinations to the initial corticosteroid therapy. Two independent researchers reviewed the data, and disagreement between them has been resolved by consensus.

### Statistical Analysis

Continuous data were summarized as median (interquartile range, IQR) or mean ± standard deviation (SD), and categorical data were summarized as frequency (percentage). The difference of continuous variables was compared using t test or nonparametric test. The difference of categorical variables was compared using Chi-square (*χ*2) or Fisher’s exact test. Overall survival (OS) was defined as the onset of myocarditis to the date of death from any cause. At the time of analysis, patients who were alive or lost follow-up were considered censored. Kaplan-Meier was used for survival analysis, and the log-rank test was used to assess differences between groups. Cox’s proportional regression analysis was conducted to identify significant variables that affect patients’ survival after myocarditis and estimate hazard ratios (HR) and 95% confidence interval (CI) for predictors of survival. All tests were two-tailed, and p < 0.05 was considered statistically significant.

## Results

### Clinical Characteristics of All Patients

The baseline characteristics of all patients are shown in **Table 1**. A total of 46 patients were enrolled in this study. All patients received anti-PD-1 treatment, and only one case was confirmed pathologically (**Supplementary Table 2**). Most of them were males, less than 60 years old and non-smokers. Patients with heart-related risk factors accounted for 54.3% (25/46), with smoking (15/46, 32.6%) predominating, followed by hypertension (14/46, 30.4%). A total of 10 types of tumors were involved in the study, with lung cancer being the most (58.7%), followed by thymoma (13.0%). The majority of patients were stage IV (67.4%) and had a history of tumor treatment (60.9%). Most of them were using a combination regimen of immunotherapy (67.4%). 87.0% of patients had significant symptoms when ICI myocarditis occurred, mainly related to heart failure, such as chest tightness (60.9%), shortness of breath (43.5%), wheezing (21.7%), myasthenia gravis (17.4%), palpitations (15.2%), and orthopnea (13.0%).Thirty patients (65.2%) developed other irAEs during immunotherapy, including hepatitis, myositis, myasthenia gravis and pneumonia, of which 70% (21/30) were coincident with myocarditis. Among all patients, the number of severe myocarditis, heart failure (grade 3-4) and myocarditis (grade 3-4) accounted for 69.6%. Notably, 5 of six thymoma patients had myasthenia gravis, and 4 of them (4/5, 80%) had concurrent myositis. Myocarditis was detected in all six patients with thymoma. Cardiac magnetic resonance imaging (MRI) was performed on 10 patients, of whom 8 had abnormal findings, including abnormal myocardial enhancement (75%, 6/8), abnormal cardiac morphology (50%, 4/8), decreased cardiac function (37.5%), 3/8), myocardial edema (25%, 2/8) and pericardial effusion (25%, 2/8) (**Supplementary Table 3**).

**Table 1:** Clinical characteristics of all patients.

Clinical characteristics	Patients (n=46)
Age (<60 years old), n (%)	35 (76.1)
BMI (mean ± SD)	
Male	22.5 ± 3.4
Female	23.1 ± 3.4
Gender, (male, %)	36 (78.3)
Risk factors related to heart disease, (yes, %)	25 (54.3)
Smoking	15 (32.6)
Hypertension	14 (30.4)
Diabetes melliitus	7 (15.2)
Coronary Heart Disease	5 (10.9)
Others	4 (8.7)
Cancer type, n (%)	
Lung cancer	27 (58.7)
Thymoma	6 (13)
Nasopharyngeal carcinoma	3 (6.5)
Liver cancer	3 (6.5)
Melanoma	2 (4.3)
Lymphoma	1 (2.2)
Kidney Cancer	1 (2.2)
Ureteral cancer	1 (2.2)
Rectal cancer	1 (2.2)
Endometrial cancer	1 (2.2)
Clinical stage	
II	2 (4.3)
III	13 (28.3)
IV	31 (67.4)
Previous treatment, (yes, %)	28 (60.9)
Immunotherapy, (combination, %)	31 (67.4)
Symptoms of myocarditis, (yes, %)	40 (87.0)
Chest tightness	28 (60.9)
Shortness of breath	20 (43.5)
Wheezing	10 (21.7)
Myasthenia gravis	8 (17.4)
Palpitations	7 (15.2)
Chest pain	7 (15.2)
Orthopnea	6 (13.0)
Muscle weakness	5 (10.9)
Fever	4 (8.7)
Blurred vision	3 (6.5)
Myalgia	3 (6.5)
Fatigue	2 (4.3)
Swelling of lower extremities	2 (4.3)
Dizziness	2 (4.3)
Multi-organ irAEs, (yes, %)	30 (65.2)
Hepatitis	15 (32.6)
Myositis	14 (30.4)
Pneumonitis	7 (15.2)
Thyroiditis	5 (10.9)
Nephritis	4 (8.7)
Dermatitis	2 (4.3)
Enteritis	1 (2.2)
Sequence of myocarditis and other irAEs(n=30)	
Simultaneous	21 (70)
Myocarditis first	2 (6.7)
Myocarditis later	7 (23.3)
Grade of heart failure	
0	5 (10.9)
2	9 (19.6)
3	12 (26.1)
4	20 (43.5)
Grade of myocarditis	
1	3 (6.5)
2	11 (23.9)
3	8 (17.4)
4	24 (52.2)
Severe myocarditis, (yes, %)	32 (69.6)

BMI, body mass index; SD, standard deviation.

### Time to Event Onset and Peripheral Blood Changes of ici Myocarditis

**Figure 1:**
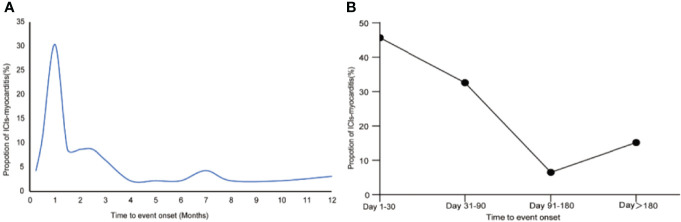
Time to event onset of ICI myocarditis. **(A)** Incidence trend by month to event onset. **(B)** Incidence trend by time period (day 1-30, day 31-90, day 91-180, day > 180).

Time-event frequency distribution is shown in **Figure 1**. We depicted the time-event distribution curve of ICI myocarditis. The results showed that immune-related myocarditis mainly occurred within 1 month after receiving ICI and 78.2% within 3 months. We compared the changes in blood test of patients at baseline and onset of myocarditis and found that in addition to the significant decrease in ALC, NLR, C-reactive protein (CRP), lactate dehydrogenase (LDH), interleukin (IL)-6, IL-10, creatine kinase (CK), MB isoenzyme of creatine kinase (CK-MB), and brain natriuretic peptide (BNP) all increased significantly (**Table 2**). ALC, NLR, LDH, IL-6, IL-10, CK, CK-MB, BNP changed more than 70% of the patients.

**Table 2:** Changes in labortory tests.

Boold test	n	Baseline	Onset of myocarditis	p	Trend	Proportion
ALC (10^9^/L)	34	1.1 (0.7-1.625)	0.6 (0.5-1.2)	0	decrease	26 (76.5)
NLR	34	3.8 (2.35-7.05)	9.8 (4.8-18.4)	0	increase	29 (85.3)
CRP (mg/L)	19	13.45 (5.35-62.225)	31.2 (8.625-94.2)	0.045	increase	12 (63.2)
LDH (U/L)	25	232.1(184.4-311.15)	613 (296-1050.8)	0	increase	21 (84.0)
IL-6 (pg/mL)	16	7.45 (3.725-12.825)	15.4 (7.075-37.375)	0.004	increase	12 (75.0)
IL-10 (pg/mL)	16	2.45 (1.475-5.0)	3.85 (2-7.45)	0.018	increase	13 (81.3)
CK (U/L)	27	63.2 (49.225-107.0)	477.2 (101.0-3214.2)	0	increase	23 (85.2)
CK-MB (U/L)	21	10.0 (2.3-14.0)	39.0 (17.0-103.45)	0	increase	19 (90.5)
BNP (pg/mL)	19	103.8 (35.85-258.25)	1704 (194.1-4848)	0	increase	19 (100.0)

ALC, absolute lymphocyte count; NLR, neutrophil to lymphocyte ratio; CRP, C-reactive protein; LDH, lactate dehydrogenase; IL-6, interleukin-6; IL-10, interleukin-10; CK, creatine kinase; CK-MB, MB isoenzyme of creatine kinase; BNP, brain natriuretic peptide.

### Comparison of Population Characteristics, Tumor Treatment History, Myocarditis Characteristics and Treatment Options in Pure Myocarditis and Multi-organ irAEs

**Table 3** shows the differences between pure myocarditis and multi-organ irAEs. There was a significant statistical difference in the classification of cancers between the two groups in comparing baseline characteristics. Compared with pure myocarditis, thymoma accounts for more multi-organ irAEs. However, there were no significant differences in age, gender, heart-related risk factors, clinical stage, previous tumor treatment, and immunotherapy. In comparing the characteristics, treatment and outcome of myocarditis, there were significant statistical differences in symptoms, treatment time after myocarditis, arrhythmia, grade of heart failure, grade of myocarditis, initial dose of corticosteroids, and severe myocarditis between the two groups. Multi-organ irAEs were prone to myocarditis-related symptoms and arrhythmia. Moreover, they were more prone to heart failure (grade 3-4) and severe myocarditis, and half of the patients received high-dose corticosteroid therapy. There was no statistically significant difference between the two groups in time to receive corticosteroid therapy, blood tests at baseline or onset of myocarditis (**Supplementary Table 4**).

**Table 3:** Characteristics of the patients with or without other irAEs.

	ICI myocarditis (n=16)	Multi-organ irAEs (n=30)	*χ*2	p
Age (<60 years old, %)	10 (62.5)	15 (50.0)	0.675	0.418
Gender (males,%)	11 (68.8)	25 (83.3)	–	0.283
Heart-related risk factors, (yes, %)	10 (65.2)	15 (50)	0.675	0.418
Classification of cancer			–	0.025
Lung cancer	8 (50.0)	19 (63.3)		
Thymoma	0	6 (20.0)		
Other cancers	8 (50.0)	5 (16.7)		
Clinical stage (n, %)			2.144	0.143
II-III	3 (18.8)	12 (40.0)		
IV	13 (81.3)	18 (60.0)		
Previous tumor treatment, (yes, %)	11 (68.8)	17 (56.7)	0.64	0.424
Immunotherapy, (combination, %)	11 (68.8)	20 (66.7)	0.021	0.886
Symptoms of myocarditis, (yes, %)	11 (68.8)	29 (96.7)	–	0.015
Arrhythmia, (yes, n, %)	4 (25.0)	20 (66.7)	7.26	0.012
Malignant arrhythmia (yes, n, %)	2 (12.5)	16 (53.3)	7.305	0.007
malignant auricular arrhythmia	1 (6.3)	8 (26.7)		
III° AV Block	1 (6.3)	8 (26.7)		
malignant ventricular arrhythmia	1 (6.3)	5 (16.7)		
Grade of heart failure (NYHA)				0.000
0-2	11 (68.8)	3 (10.0)		
3-4	5 (31.3)	27 (90.0)		
Grade of myocarditis				0.000
1-2	11 (68.8)	3 (10.0)		
3-4	5 (31.3)	27 (90.0)		
Corticosteroid therapy, (yes, %)	14 (87.5)	29 (96.7)		0.247
Time to receive corticosteroid therapy (n=43)			0.096	1.000
Within 24 hours	6 (42.9)	11 (37.9)		
Over 24 hours	8 (57.1)	18 (62.1)		
Initial corticosteroid therapy			4.669	0.031
High Dose	2 (14.3)	14 (48.3)		
Low Dose	12 (85.7)	15 (51.7)		
Severe myocarditis, (yes, %)	5 (31.3)	27 (90.0)	–	0.000

NYHA, New York Heart Association.

### Survival Analysis and Prognostic Factors in All Patients

**Figure 2:**
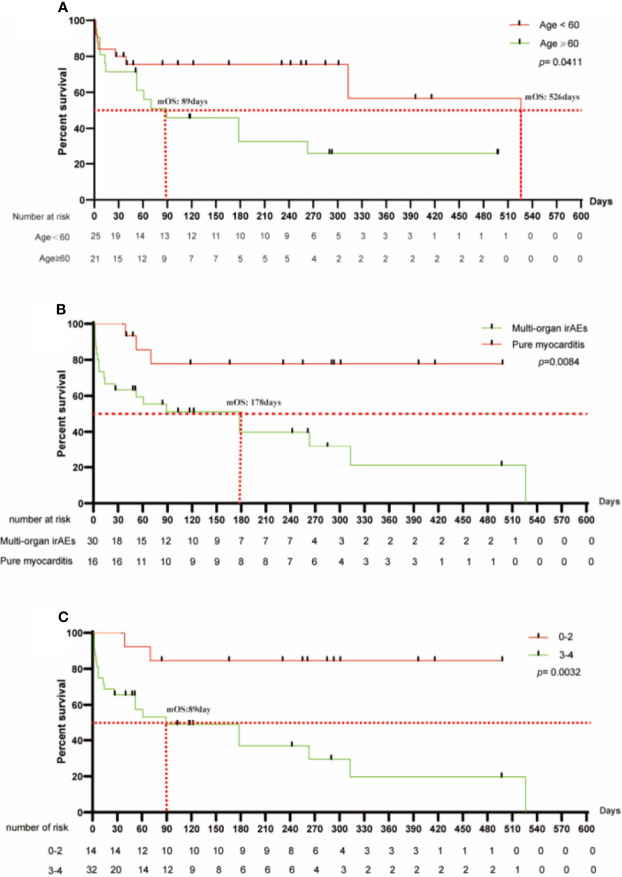
Survival analysis of ICI myocarditis by stratifications. **(A)** In stratification of age, the mOS of the <60 group was significantly longer than that of the ≥60 group (526d versus 89d, p=0.041). **(B)** In the stratification of irAEs, multi-organ irAEs have a worse prognosis than pure myocarditis, with an mOS of 178d and not reached. **(C)** In the stratification of heart failure, heart failure (grade 3-4) has a worse prognosis, with an mOS of 89d.

All patients were followed up until September 2021. Six of the 46 patients were lost to follow-up, and 18 were still alive. The median follow-up time was 261 days and the median OS (mOS) was 263 days. Among the deaths, the causes of death were myocarditis (11/22, 50%), tumor (6/22, 27.3%) and CIP (5/22, 22.7%) (**Supplementary Table 5**). Analysis of prognostic factors was performed in all patients to determine the important variables that affect OS after myocarditis (**Table 4**). Univariate analysis showed that patients with age ≥60 years, myocarditis (grade 3-4), heart failure (grade 3-4), multi-organ irAEs, and severe myocarditis have a worse prognosis (**Figure 2**, **Supplementary Table 5**). We found that the survival analysis results were consistent in myocarditis grade (grade 3-4), heart failure grade (grade 3-4) and severe myocarditis. Incorporating the variables with p<0.1 in the univariate analysis into the multivariate analysis model, we found that myocarditis grade was linearly subordinate to the heart failure grade. Further analysis showed that after adjusting for age and other irAEs in all patients, heart failure (grade 3-4) was an independent risk factor for immune-related myocarditis (HR: 6.655, 95% CI: 1.539-28.770, *p*=0.011). In the stratification of heart failure grade, the mOS of patients with heart failure (grade 3-4) was 178 days, and1-month and 3-month OS rate was 60% and 40%, respectively. While mOS of patients with heart failure (grade 0-2) was not reached, and1-month and 3-month OS rate was 100% and 62.5%, respectively.

**Table 4:** Univariate and multivariate analysis of prognostic factors in all patients.

	No. patients (%)	Overall survival	
		Univariate analysis: P	Multivariate analysis: P
Age (<60 years old, %)	35 (76.1)	0.041	0.297
Gender (males,%)	36 (78.2)	0.403	–
Heart-related risk factors (yes, %)	25 (54.3)	0.094	0.127
Clinical stage (IV, %)	31 (67.4)	0.831	–
Previous treatment, (yes, %)	28 (60.9)	0.247	–
Immunotherapy, (combination, %)	31 (67.4)	0.785	–
Symptoms of immune-related myocarditis, (yes, %)	40 (87.0)	0.104	0.394
Arrhythmia, (yes, %)	24 (52.2)	0.577	–
Heart failure grade (Grade 3-4,%)	32 (69.6)	0.003	0.011
Myocarditis grade (Grade 3-4,%)	32 (69.6)	0.003	*
Multi-organ irAEs, (yes, %)	30 (65.2)	0.008	0.29
Severe myocarditis, (yes, %)	32 (69.6)	0.003	0.561
Initial corticosteroid therapy (high dose)	16 (37.2)	0.779	–

*Grade of myocarditis are linearly subordinate to the grade of heart failure.

**Figure 3:**
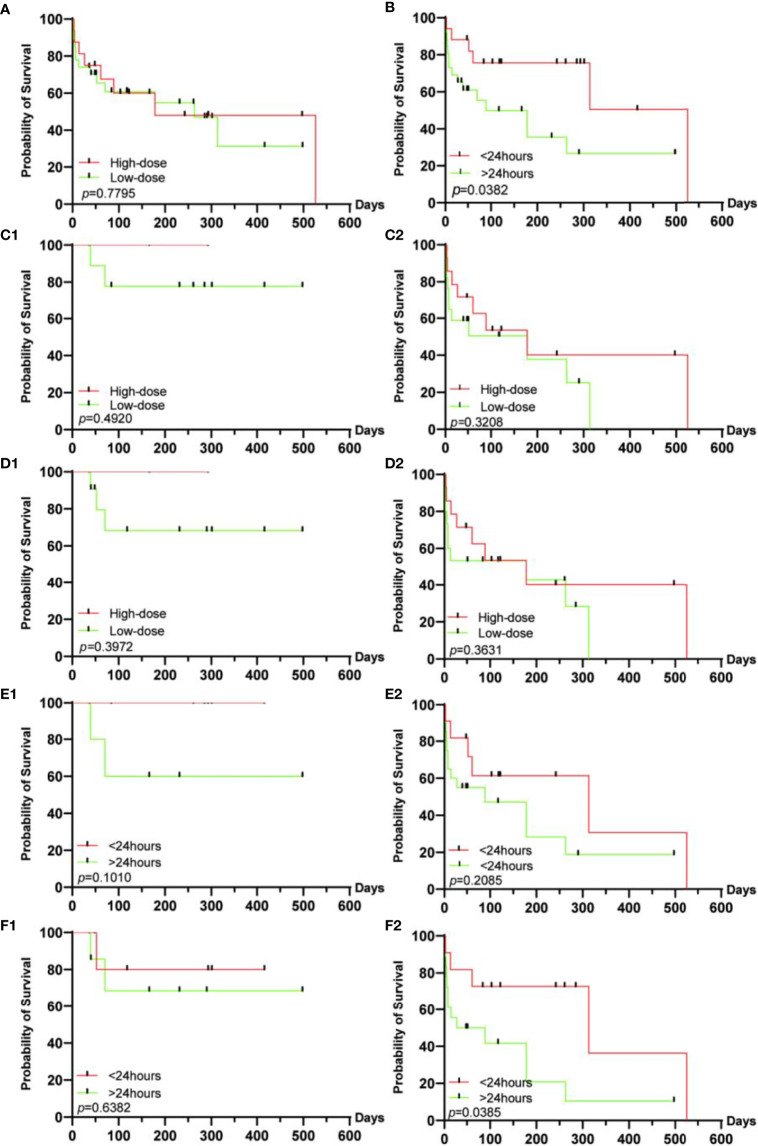
Survival analysis of corticosteroid therapy in different groups. **(A)** In all patients, the dose of corticosteroids has no significant effect on survival, **(B)** while early corticosteroid intervention can improve the prognosis. Regardless of the heart failure grade [0-2 **(C1)**, 3-4 **(C2)**] or the pattern of organ involvement [pure myocarditis **(D1)**, multi-organ irAEs **(D2)**], the dose of corticosteroids has no significant effect on the prognosis. Regardless of the grade 0-2 **(E1)** or the grade 3-4 **(E2)** of heart failure, the intervention time of corticosteroids have no significant effect on the prognosis. **(F1)** In pure myocarditis, the duration of hormonal intervention has no significant effect on the prognosis, **(F2)** while in patients with multiple organ irAEs, early hormonal intervention can significantly improve the prognosis of patients.

In addition, we explored the population characteristics and prognosis of multi-organ irAEs with or without myositis. But there were no statistically significant differences in mortality, higher-grade arrhythmias, or greater degree of myocarditis between the 2 subgroups (**Supplementary Tables 5, 6**). The effect of corticosteroid therapy on prognosis was explored in all patients (**Figure 3**). We found that the dose of corticosteroids had no effect on survival, but earlier corticosteroid therapy (<24 hours) resulted in better survival. Furthermore, we explored the efficacy of corticosteroids usage in different grades of heart failure and organ involvement mode. We found that therewas no significant difference in the onset time of corticosteroids in different grades of heart failure. In the stratification of organ involvement patterns, we found that earlier hormonal intervention has a better prognosis when multiple organs were involved. However, there was no difference in the comparison of corticosteroid usage in the two strata.

## Discussion

In the present study, we investigated the impact of multi-organ irAEs on ICI-associated myocarditis and some the risk factors. We found that more than half of ICI myocarditis patients were multi-organ irAEs, and the presence of multi-organ adverse events and severe myocarditis lead to reduced overall survival in patients with ICI induced myocarditis.

Up to date, it seems impossible to predict which patients could be affected by myocarditis after ICI treatment. To our knowledge, there are currently no effective approaches or guidelines to predict ICI-associated cardiotoxicity. Our study was partially designed as a nested case-control study in a cohort comprising ICI myocarditis patients with or without other irAEs. Since this study did not include non-myocarditis patients, it was not possible to predict myocarditis events in patients receiving ICI.

Consistent with previous studies, male sex, heart-related risk factors and the combined therapy are more common in patients with myocarditis, and most of them have a history of tumor treatment (6, 16). However, inconsistent with previous studies, most patients in our study were less than 60 years old, non-smokers, had no heart-related risk factors and lower BMI (6, 9, 17). Highly similar to previous studies, our study showed that most ICI myocarditis occurred within 1 month of receiving ICIs treatment, and the incidence within 3 months was 78.2% (5). Most patients with myocarditis have obvious symptoms, mainly related to heart failure. When combined with multi-organ irAEs, the incidence of symptoms is as high as 96.7%. A study showed that the incidence of MACE in patients receiving glucocorticoid therapy within 24 hours after admission for myocarditis was lower than that of patients receiving glucocorticoid therapy within 24-72 hours and >72 hours (18). Therefore, although symptoms such as ICI myocarditis are not specific, timely medical treatment and corticosteroid therapy after symptoms appear can reduce the incidence of MACE. The effect of multi-organ irAEs on ICI myocarditis is rarely reported. Our research found that age, gender, smoking history, heart disease-related risk factors, tumor treatment history, immunotherapy regimen are not risk factors for multi-organ irAEs in patients with myocarditis. It is worth noting that all 6 thymoma patients (B2/B3) in this study had multi-organ irAEs, and 2 died of myocarditis. Previous studies have reported that patients with thymoma or thymic carcinoma are prone to multi-organ involvement such as myocarditis, myositis, and hepatitis when receiving ICIs (19–21). Myasthenia gravis (MG), myositis, and myocarditis overlap syndrome is a potentially fatal adverse event of ICIs (22). Johnson et al. found the same selective clonal T-cell populations infiltrating in the myocardium, skeletal muscle and tumors in 2 patients with fatal myocarditis and concomitant myositis (7). In addition, they found high levels of muscle-specific antigens in the tumors of these patients, suggesting that activated T lymphocytes after immunotherapy may attack cardiomyocytes and muscle cells through shared epitopes, leading to the development of autoimmune myocarditis and myositis. Thymoma patients receiving ICIs are prone to develop MG, myositis and myocarditis. Cho et al. reported that 3 cases of 7 patients with thymoma developed grade 3 myocarditis (42.9%), and these patients were of type B2 or B3 (19). In our study, all thymoma patients had grade 3-4 myocarditis. Moreover, 5 of 6 thymoma patients developed MG, and 4 of them (80%) had concurrent myositis. All these suggest that type B2 or B3 thymoma is more prone to severe irAEs. Furthermore, multi-organ irAEs have higher grade of heart failure and myositis, more malignant arrhythmias, and more high-dose corticosteroid therapy. The above evidence shows that multi-organ irAEs in ICI myocarditis are prone to become severe myocarditis. Further survival analysis showed that patients with multi-organ irAEs have a worse prognosis with an mOS of 178 days.

So far, few studies have analyzed the risk of survival after ICI myocarditis. Previous studies have shown that ALC reduction>35%, NLR increase>100% and extended duration of QRS are risk factors for MACE (10, 11). In our study, we found that multi-organ irAEs, age≥60 years old, myocarditis (G3-4), heart failure (G3-4), and severe myocarditis are risk factors on survival for ICI myocarditis. However, further multivariate analysis found that the grade of myocarditis have a linear relationship with the grade of heart failure. The possible reason is that in our study, the evaluation criteria of the grade of myocarditis were similar to the grade of heart failure. After adjusting for other factors, we found that the severity of heart failure is an independent risk factor that affects the survival of patients after myocarditis. The above results suggest that the classification of heart failure can be used as risk stratification for ICI myocarditis.

Whether multi-organ irAEs can be used as risk stratification factors for ICI myocarditis is worth discussing. As mentioned above, patients with other irAEs are more prone to severe heart failure. Heart failure after ICI myocarditis is caused by various mechanisms, including blockade of immune checkpoints of the myocardium, accumulation of cytokines, and microvasculitis of the heart (23, 24). In ICI myocarditis, the increased circulating cytokine levels aggravate the damage of cardiomyocytes (23, 25), which may further aggravate heart failure. Our study found that compared with baseline, cytokines increased significantly after the occurrence of irAE, which is consistent with previous studies (10, 26). However, it has not been reported whether multi-organ irAEs have higher cytokines than single-organ irAEs. Our study found no difference in cytokine levels between pure myocarditis and multi-organ irAEs when myocarditis occurs. Considering the limited patients, a large sample size study is needed to explore the difference in cytokines between single-organ and multi-organ irAEs. In view of the above evidence, we cannot explain the mechanism of the effect of multi-organ involvement on severe heart failure. However, due to the strong correlation between multi-organ irAEs and more severe heart failure, we believe multi-organ irAEs can be used as risk stratification factors in ICI myocarditis.

The administration of corticosteroids plays an important role in improving the prognosis of irAEs (15, 27). For the timimg and dose of corticosteroids in ICI myocarditis, the current exploration is still limited. Zhang et al. have shown that high-dose and early corticosteroid intervention (<24 hours) can significantly reduce the occurrence of MACE (18). In our study, we found that earlier corticosteroid intervention but not high-dose corticosteroids improved the prognosis of patients. Further stratified exploration found that patients with multi-organ irAEs benefited from earlier corticosteroid intervention. It is further noted that the timing of corticosteroid therapy in the study is from the appearance of heart-related symptoms or asymptomatic but abnormal heart examination to the initial corticosteroid therapy, while Zhang et al. is from the diagnosis of myocarditis to the initial corticosteroid therapy. Our research shows that receiving corticosteroid treatment within 24 hours after the appearance of heart-related symptoms or abnormal tests is the golden treatment period, especially for patients with severe myocarditis. In other words, this suggests that doctors should pay attention to patient education, such as emphasizing the early symptoms of myocarditis in the informed consent, so that the patient can contact the specialist or attending physician for diagnosis or elimination as soon as possible. For patients with multi-organ irAEs, who are prone to severe heart failure and worse prognosis, high-dose corticosteroids that reduce MACE should be the first choice. Multidisciplinary consultations should be conducted to develop an individualized treatment plan.

Several limitations need to be noted regarding this study. First of all, this is a retrospective study with a small sample. The lack of blood tests makes it difficult to compare the differences between patients with pure myocarditis and multi-organ irAEs. Secondly, this study involved a variety of tumors, and the internal heterogeneity of the samples was large. Finally, a short follow-up time and too many censored events lead to poor stability of survival analysis results. Further research with a larger patient population are needed to confirm our findings.

## Conclusions

Patients with ICI-associated myocarditis had multi-organ irAEs with a high incidence of severe myocarditis, mortality, and poor prognosis. Thymoma (B2/B3) was prone to those patients with multiple organs involvement. Patients cound benefit from early corticosteroid intervention (<24 hours). Heart failure (grade 3-4)was an independent risk factor for OS after myocarditis.

## Data Availability Statement

The original contributions presented in the study are included in the article/**Supplementary Material**. Further inquiries can be directed to the corresponding author.

## Ethics Statement

Institutional review board/ethics committee approval was obtained from the Institutional Review Board of the First Affiliated Hospital of Guangzhou Medical University (Guangzhou, Guangdong, China). Individual consent for this retrospective analysis was waived.

## Author Contributions

(I) Conception and design: XX, LW, YX, YL, CZ; (II) Provision of study materials or patients: YQ, MjL, XL, WL, ZC, QM, ZX, JZ, YL, CZ; (III) Collection and assembly of data: LW, JW; (IV) Data analysis and interpretation: XX, LW, YX, XL, YL; ML; BL (V) Manuscript writing: XX, LW; (VI) Final approval of manuscript: All authors.

## Funding

Guangdong Science and Technology Program special projects [2020A1111350025], Fundamental and Applied Fundamental Research Project of City-School (Institute) Joint Funding Project, Guangzhou Science and Technology Bureau[202102010345], Zhongnanshan Medical Foundation of Guangdong Province [ZNSA-2020003] and State Key Laboratory of Respiratory Disease-The Independent project[SKLRD-Z-202117].

## Conflict of Interest

The authors declare that the research was conducted in the absence of any commercial or financial relationships that could be construed as a potential conflict of interest.

## Publisher’s Note

All claims expressed in this article are solely those of the authors and do not necessarily represent those of their affiliated organizations, or those of the publisher, the editors and the reviewers. Any product that may be evaluated in this article, or claim that may be made by its manufacturer, is not guaranteed or endorsed by the publisher.
