# Supplementary material for: Multi-organ Immune-Related Adverse Event Is a Risk Factor of Immune Checkpoint Inhibitor-Associated Myocarditis in Cancer Patients: A Multi-center Study
Source: Front Immunol. 2022 Jul 18;13:879900. doi: 10.3389/fimmu.2022.879900 (PMC9340212; doi:10.3389/fimmu.2022.879900)
Supplement: Supplementary file 1 [file DataSheet_1.docx]

**Multi-organ immune-related adverse event is a risk factor of immune checkpoint inhibitor-associated myocarditis in cancer patients: a multi-center study**

**SUPPLEMENTARY MATERIALS**

**Table of Contents**

**Supplementary Table 1.** Medical centers and number of cases......…….…**…**…….……...3

**Supplementary Table 2. The kinds of ICI and diagnosis of myocarditis & myositis..........4**

**Supplementary Table 3. Features of cardiac MRI...............................................................5**

**Supplementary Table 4. Blood tests on baseline and onset of myocarditis*…..........*……...6**

**Supplementary Table 5. Survival analysis and cause of death analysis by stratification of age, irAEs, grade of myocarditis, grade of heart failure, severe disease, and multi-organ irAEs with or without myocarditis.……......................................................................…...7**

**Supplementary Table 6. Characteristics of the in multi-organ irAEs patients with or without** myositis**..................................................................................................................8**

**Supplementary Table 7. Partial clinical data of all** cases**....................................................9**

**Supplementary Table 1. Medical centers and number of cases.**

| **Medical centers** | **Number of myocarditis cases** |
| --- | --- |
| **The First Affiliated Hospital of Guangzhou Medical University** | **18** |
| **Sun Yat-sen University Cancer Center** | **10** |
| **Peking Union Medical College Hospital** | **13** |
| **Cancer Hospital Of Shantou University Medical College** | **2** |
| **Cancer Center of Guangdong Provincial People’s Hospital** | **1** |
| **The Third Affiliated Hospital of Sun Yat-sen University** | **2** |

**Supplementary Table 2. The kinds of ICI and diagnosis of myocarditis & myositis.**

|  | **Number of cases** |
| --- | --- |
| **The kinds of ICIs (n=46)** | **46** |
| **Pembrolizumab** | **16** |
| **Sintilimab** | **15** |
| **fTislelizumab** | **5** |
| **Camrelizumab** | **5** |
| **Toripalimab** | **3** |
| **Nivolumab** | **2** |
| **Diagnosis of myocarditis (n=46)** |  |
| **Histopathology** | **1** |
| **Clinically suspected myocarditis** | **45** |
| **Diagnosis of myositis (n=14)** |  |
| **Muscle biopsy (if available)** | **1** |
| **Clinically confirmed**  **(Clinical diagnosis (elevated CK and/or myositis antibody profile and/or myalgia and/or muscle weakness))** | **13** |
| **Muscle electrogram (if available)** | **0** |
| **Muscle MRI (if available)** | **0** |

|  | |
| --- | --- |
| Features of cardiac MRI | (N=10) |
| Normal | 3 |
| myocardial delayed enhancement | 6 |
| Abnormal cardiac morphology | 4 |
| Decreased cardiac function | 3 |
| Myocardial edema | 2 |
| Pericardial effusion | 2 |

**Supplementary Table 3.** Features of cardiac MRI.

**Supplementary Table 4. Blood tests on baseline and onset of myocarditis.**

| **Table 4A.** Baseline | | | | | |
| --- | --- | --- | --- | --- | --- |
| **Blood tests** | **Pure myocarditis** | | **Multi-organ irAEs** | | **p** |
|  | **n** | **Median (IQR)** | **n** | **Median (IQR)** |  |
| ALC (10^9^/L) | 14 | 0.9(0.675-1.275) | 20 | 1.4(0.85-1.85) | 0.145 |
| NLR | 14 | 5.3(2.4-7.5) | 20 | 3.5(2.25-5.35) | 0.296 |
| CRP (mg/L) | 11 | 8.7(4.4-65.5) | 13 | 17.5(7.75-70.05) | 1 |
| LDH (U/L) | 10 | 264.35(207.65-433.775) | 18 | 217.5(163.0-270.95) | 0.695 |
| IL-6 (pg/mL) | 6 | 6.8(4.325-11.375) | 12 | 7.7(2.775-15.075) | 1 |
| IL-10 (pg/mL) | 6 | 2.65(1.275-5.0) | 12 | 2.45(1.525-5.225) | 1 |
| CK (U/L) | 11 | 59.0(39.7-107.0) | 17 | 71.0(50.55-134.0) | 0.44 |
| CK-MB (U/L) | 8 | 10.95(0.2-13.075) | 15 | 10(8.0-15.0) | 1 |
| BNP (pg/mL) | 7 | 133(48-290) | 14 | 73.35(32.35-255.88) | 0.659 |

| ALC: absolute lymphocyte count; NLR: neutrophil to lymphocyte ratio; CRP: C-reactive protein; LDH: lactate dehydrogenase; IL-6: Interleukin-6; IL-10: Interleukin-10; CK: Creatine Kinase; CKMB: MB isoenzyme of creatine kinase; BNP: Brain natriuretic peptide |
| --- |

|  |
| --- |

| **Table 4B.** Onset of myocarditis | | | | | |
| --- | --- | --- | --- | --- | --- |
| **Blood tests** | **Pure myocarditis** | | **Multi-organ irAEs** | | **p** |
|  | **n** | **Median (IQR)** | **n** | **Median (IQR)** |  |
| ALC (10^9^/L) | 15 | 0.8(0.3-1.2) | 28 | 0.6(0.5-1.275) | 0.911 |
| NLR | 15 | 6.4(5.2-17.5) | 28 | 12.3(4.8-21.325) | 0.597 |
| CRP (mg/L) | 11 | 27.4(4.2-93.7) | 23 | 31.4(9.9-95.7) | 1 |
| LDH (U/L) | 10 | 376.0(277.425-775.2) | 25 | 747.0(401.5-1666.0) | 0.31 |
| IL6 (pg/mL) | 9 | 26.5(13.6-37.6) | 15 | 12.8(3.5-38.5) | 0.4 |
| IL10 (pg/mL) | 9 | 3.3(1.6-9.25) | 15 | 4.1(1.9-7.6) | 1 |
| CK (U/L) | 13 | 283(81.0-822.65) | 30 | 1074(102.72-10861) | 0.219 |
| CK-MB (U/L) | 13 | 23.9(8.6-42.7) | 28 | 53.9(18.35-126.075) | 0.216 |
| BNP (pg/mL) | 15 | 561.0(11.8-4325.0) | 28 | 2601.5(564.0-5325.9) | 0.597 |
| ALC: absolute lymphocyte count; NLR: neutrophil to lymphocyte ratio; CRP: C-reactive protein; LDH: lactate dehydrogenase; IL-6: Interleukin-6; IL-10: Interleukin-10; CK: Creatine Kinase; CKMB: MB isoenzyme of creatine kinase; BNP: Brain natriuretic peptide | | | | | |

**Supplementary Table 5. Survival analysis and cause of death analysis by stratification of age, irAEs, grade of myocarditis, grade of heart failure, severe disease, and multi-organ irAEs with or without myocarditis.**

|  | | | | | | | | | | |
| --- | --- | --- | --- | --- | --- | --- | --- | --- | --- | --- |
| **Groups** | **Stratification** | **n** | **Events** | **Censored** | **mOS (days)** | **HR (95%CI)** | **Cause of Death** | | |  |
|  |  |  |  |  |  |  | **Myocarditis** | **Tumor** | **CIP** | **p** |
| **All** |  | 46 | 22 | 24 | 263 |  | 11 | 6 | 5 | - |
| **Age** |  |  |  |  |  |  |  |  |  | 0.304 |
|  | <60 | 25 | 8 | 17 | 526 | 0.4262 (0.1831 to 0.9920) | 6 | 1 | 1 |  |
|  | >60 | 21 | 14 | 7 | 89 | Reference | 5 | 5 | 4 |  |
| **Heart-related risk factors** |  |  |  |  |  |  |  |  |  |  |
|  | Yes | 19 | 7 | 12 | 526 | 0.3835 (0.1593 to 0.9230) |  |  |  |  |
|  | No | 27 | 15 | 12 | 178 | Reference |  |  |  |  |
| **Multi-organ irAEs** |  |  |  |  |  |  |  |  |  | 0.019 |
|  | Yes | 30 | 19 | 11 | 178 | 4.287 (1.829 to 10.05) | 11 | 3 | 5 |  |
|  | No | 16 | 3 | 13 | NR | Reference | 0 | 3 | 0 |  |
| **Grade of Heart Failure** |  |  |  |  |  |  |  |  |  | 0.108 |
|  | 0-2 | 14 | 2 | 12 | NR | 0.1594 (0.0676 to 0.3762) | 0 | 2 | 0 |  |
|  | 3-4 | 32 | 20 | 12 | 89 | Reference | 11 | 4 | 5 |  |
| **Grade of Myocarditis** |  |  |  |  |  |  |  |  |  | \| 0.108 \| \| --- \| |
|  | 1-2 | 14 | 2 | 12 | NR | 0.1594 (0.0676 to 0.3762) | 0 | 2 | 0 |  |
|  | 3-4 | 32 | 20 | 12 | 89 | Reference | 11 | 4 | 5 |  |
| **Severe myocarditis** |  |  |  |  |  |  |  |  |  | 0.108 |
|  | No | 14 | 2 | 12 | NR | 0.1594 (0.0676 to 0.3762) | 0 | 2 | 0 |  |
|  | Yes | 32 | 20 | 12 | 89 | Reference | 11 | 4 | 5 |  |
| **Multi-organ irAEs with myocarditis** |  |  |  |  |  |  |  |  |  | 0.104 |
|  | Yes | 14 | 6 | 8 | 178 | 0.481 (0.178 to 1.299) | 4 | 2 | 0 |  |
|  | No | 16 | 13 | 3 | 14 | Reference | 7 | 1 | 5 |  |

**Supplementary Table 6.** Characteristics of the in multi-organ irAEs patients with or without myositis.

|  | | | | |
| --- | --- | --- | --- | --- |
|  | Patients with myositis | | *χ*2 | p |
|  | Yes (N=14) | No (N=16) |  |  |
| Age (<60 years old, %) | 7(50%) | 8(50%) | 0 | 1 |
| Gender (males, %) | 11(78.6%) | 14(87.5%) | - | 0.642 |
| Smoking status (yes, %) | 4(28.6%) | 6(37.5%) | - | 0.709 |
| Heart-related risk factors (yes, %) | 5(35.7%) | 6(37.5%) | 0.01 | 1 |
| Clinical stage (IV, %) | 10(71.4%) | 8(50%) | 1.429 | 0.284 |
| Cancer type, n (%) |  |  | - | 0.155 |
| Lung cancer | 7(50%) | 12(75%) |  |  |
| Thymoma | 5(35.7%) | 1(6.3%) |  |  |
| Other cancers | 2(14.3%) | 3(18.8%) |  |  |
| Previous treatment, (yes, %) | 8(57.1%) | 9(56.3%) | 0.002 | 1 |
| Immunotherapy, (combination, %) | 8(57.1%) | 12(75%) | 1.071 | 0.442 |
| Malignant arrhythmia, (yes, %) | 9(64.3%) | 7(43.8%) | 1.265 | 0.299 |
| Heart failure grade (Grade 3-4, %) | 13(92.9%) | 14(87.5%) | - |  |
| Myocarditis grade (Grade 3-4, %) | 13(92.9%) | 14(87.5%) | - | 1 |

| **Supplementary Table 7. Partial clinical data of all c**ases. | | | | | | | | | | | | | | | | | | | | |
| --- | --- | --- | --- | --- | --- | --- | --- | --- | --- | --- | --- | --- | --- | --- | --- | --- | --- | --- | --- | --- |
| **Patient**  **No.** | | **Sex** | | **Age** | | **Heart-related risk factors** | | **Cancer type** | | **Previous treatment** | | **Immunotherapy** | | | **Combined therapy** | **Grade of myocarditis** | | **Sequential order of myocarditis and other irAEs** | **OS (days)** | **Status** |
| 1 | M | | 57 | | No | | NSCLC | | No | | Sintilimab | | PD-1 | Pemetrexed + Carboplatin | | | 4 | Concurrent | 2 | Dead |
| 2 | M | | 71 | | No | | NSCLC | | No | | Pembrolizumab | | PD-1 | - | | | 4 | Concurrent | 7 | Dead |
| 3 | M | | 63 | | No | | NSCLC | | Yes | | Sintilimab | | PD-1 | - | | | 4 | Concurrent | 61 | Dead |
| 4 | M | | 69 | | No | | NSCLC | | No | | Pembrolizumab | | PD-1 | Pemetrexed + Carboplatin | | | 4 | Myocarditis first | 178 | Dead |
| 5 | F | | 50 | | No | | NSCLC | | Yes | | Pembrolizumab | | PD-1 | Gemcitabine + Nedaplatin | | | 4 | Concurrent | 122 | Alive or Censored |
| 6 | M | | 79 | | Yes | | NSCLC | | Yes | | Pembrolizumab | | PD-1 | Bevacizumab | | | 4 | Concurrent | 89 | Dead |
| 7 | F | | 66 | | No | | NSCLC | | Yes | | Sintilimab | | PD-1 | Gemcitabine + Recombinant human endostatin | | | 4 | NA | 52 | Dead |
| 8 | F | | 52 | | No | | Thymoma | | Yes | | Sintilimab | | PD-1 | Epirubicin + Cyclophosphamide + Cisplatin | | | 4 | Myocarditis later | 242 | Alive or Censored |
| 9 | M | | 64 | | Yes | | NSCLC | | Yes | | Sintilimab | | PD-1 | Pemetrexed + Cisplatin | | | 4 | Concurrent | 51 | Alive or Censored |
| 10 | M | | 58 | | No | | Thymoma | | Yes | | Sintilimab | | PD-1 | Paclitaxel Liposome + Carboplatin | | | 4 | Concurrent | 4 | Dead |
| 11 | M | | 58 | | No | | NSCLC | | No | | Camrelizumab | | PD-1 | Albumin Paclitaxel + Carboplatin | | | 1 | NA | 36 | Alive or Censored |
| 12 | M | | 53 | | No | | Lymphoma | | Yes | | Sintilimab | | PD-1 | Lenalidomide | | | 3 | Myocarditis later | 5 | Dead |
| 13 | M | | 53 | | Yes | | Kidney cancer | | Yes | | Toripalimab | | PD-1 | Cabozantinib | | | 2 | NA | 301 | Alive or Censored |
| 14 | M | | 73 | | Yes | | NSCLC | | Yes | | Pembrolizumab | | PD-1 | Pemetrexed + Carboplatin + Bevacizumab | | | 2 | NA | 293 | Alive or Censored |
| 15 | M | | 46 | | No | | Melanoma | | Yes | | Pembrolizumab | | PD-1 | Dacarbazine | | | 2 | NA | 84 | Alive or Censored |
| 16 | M | | 36 | | Yes | | NPC | | Yes | | Toripalimab | | PD-1 | Albumin Paclitaxel + Cisplatin + Capecitabine | | | 2 | Concurrent | 285 | Alive or Censored |
| 17 | M | | 36 | | No | | Rectal cancer | | No | | Sintilimab | | PD-1 | Oxaliplatin + Capecitabine | | | 2 | Concurrent | 261 | Alive or Censored |
| 18 | M | | 40 | | No | | NPC | | Yes | | Camrelizumab | | PD-1 | Albumin paclitaxel + Capecitabine + Apatinib | | | 2 | NA | 255 | Alive or Censored |
| 19 | F | | 52 | | No | | Endometrial cancer | | Yes | | Sintilimab | | PD-1 | Fruquintinib | | | 2 | NA | 231 | Alive or Censored |
| 20 | F | | 49 | | No | | NPC | | Yes | | Toripalimab | | PD-1 | Albumin Paclitaxel + Cisplatin | | | 2 | NA | 166 | Alive or Censored |
| 21 | M | | 53 | | Yes | | Melanoma | | No | | Camrelizumab | | PD-1 | Dacarbazine | | | 3 | NA | 40 | Alive or Censored |
| 22 | M | | 59 | | No | | NSCLC | | Yes | | Nivolumab | | PD-1 | - | | | 4 | Concurrent | 26 | Dead |
| 23 | M | | 72 | | Yes | | NSCLC | | Yes | | Nivolumab | | PD-1 | - | | | 4 | Concurrent | 52 | Dead |
| 24 | M | | 58 | | Yes | | NSCLC | | No | | Pembrolizumab | | PD-1 | Albumin Paclitaxel + Carboplatin | | | 4 | Concurrent | 526 | Dead |
| 25 | M | | 80 | | Yes | | LC | | Yes | | Sintilimab | | PD-1 | - | | | 4 | NA | 290 | Alive or Censored |
| 26 | M | | 73 | | No | | NSCLC | | Yes | | Pembrolizumab | | PD-1 | - | | | 4 | Concurrent | 178 | Dead |
| 27 | M | | 64 | | Yes | | NSCLC | | Yes | | Pembrolizumab | | PD-1 | - | | | 4 | Myocarditis first | 497 | Alive or Censored |
| 28 | F | | 48 | | No | | NSCLC | | Yes | | Sintilimab | | PD-1 | Albumin Paclitaxel + Carboplatin | | | 2 | NA | 39 | Dead |
| 29 | M | | 52 | | No | | NSCLC | | Yes | | Pembrolizumab | | PD-1 | Pemetrexed + Carboplatin | | | 3 | Myocarditis later | 313 | Dead |
| 30 | M | | 66 | | Yes | | NSCLC | | Yes | | Pembrolizumab | | PD-1 | - | | | 3 | Concurrent | 263 | Dead |
| 31 | F | | 52 | | Yes | | NSCLC | | No | | Pembrolizumab | | PD-1 | Docetaxel | | | 1 | NA | 396 | Alive or Censored |
| 32 | M | | 65 | | Yes | | NSCLC | | Yes | | Sintilimab | | PD-1 | - | | | 2 | NA | 498 | Alive or Censored |
| 33 | M | | 57 | | No | | NSCLC | | Yes | | Tislelizumab | | PD-1 | - | | | 1 | NA | 416 | Alive or Censored |
| 34 | M | | 75 | | Yes | | Ureteral cancer | | No | | Tislelizumab | | PD-1 | Gemcitabine + Cisplatin | | | 3 | Concurrent | 117 | Alive or Censored |
| 35 | M | | 76 | | Yes | | LC | | No | | Camrelizumab | | PD-1 | - | | | 2 | NA | 70 | Dead |
| 36 | M | | 69 | | Yes | | LC | | No | | Camrelizumab | | PD-1 | - | | | 3 | NA | 118 | Alive or Censored |
| 37 | M | | 63 | | Yes | | NSCLC | | Yes | | Pembrolizumab | | PD-1 | Docetaxel | | | 4 | Myocarditis later | 14 | Dead |
| 38 | F | | 27 | | No | | Thymoma | | No | | Pembrolizumab | | PD-1 | Albumin Paclitaxel + Carboplatin | | | 3 | Concurrent | 2 | Dead |
| 39 | M | | 73 | | Yes | | Small cell lung cancer | | No | | Sintilimab | | PD-1 | Pemetrexed + Irinotecan + Nedaplatin | | | 4 | Myocarditis later | 7 | Dead |
| 40 | M | | 75 | | No | | NSCLC | | No | | Pembrolizumab | | PD-1 | Albumin Paclitaxel + Carboplatin + Recombinant human endostatin | | | 4 | Myocarditis later | 13 | Dead |
| 41 | M | | 74 | | No | | NSCLC | | No | | Sintilimab | | PD-1 | - | | | 4 | Myocarditis later | 2 | Dead |
| 42 | M | | 55 | | No | | Thymoma | | Yes | | Tislelizumab | | PD-1 | - | | | 4 | Concurrent | 48 | Alive or Censored |
| 43 | F | | 59 | | Yes | | Thymoma | | No | | Tislelizumab | | PD-1 | Pemetrexed + Carboplatin | | | 4 | Concurrent | 103 | Alive or Censored |
| 44 | F | | 57 | | No | | Thymoma | | No | | Sintilimab | | PD-1 | - | | | 4 | Concurrent | 27 | Alive or Censored |
| 45 | M | | 69 | | No | | NSCLC | | No | | Pembrolizumab | | PD-1 | Irinotecan + Nedaplatin | | | 4 | Concurrent | 3 | Dead |
| 46 | M | | 53 | | No | | NSCLC | | Yes | | Tislelizumab | | PD-1 | Albumin Paclitaxel + Carboplatin | | | 3 | NA | 48 | Alive or Censored |

M, male; F, female; NSCLC, non-small cell lung cancer; NPC, nasopharyngeal carcinoma; LC, liver cancer.
